# 单孔胸腔镜：微创肺癌切除的再次升华

**DOI:** 10.3779/j.issn.1009-3419.2014.07.04

**Published:** 2014-07-20

**Authors:** 成武 刘, 伦旭 刘

**Affiliations:** 610041 成都，四川大学华西医院胸外科 Department of Thoracic Surgery, West China Hospital, Sichuan University, Chengdu 610041, China

**Keywords:** 肺肿瘤, 电视胸腔镜手术, 手术, Lung neoplasms, Video-assisted thoracic surgery, Surgery

## Abstract

以电视胸腔镜手术（video-assisted thoracic surgery, VATS）为代表的微创手术已成为肺癌切除的主流。传统多孔VATS（包括四孔、三孔及两孔）已覆盖了几乎所有肺癌切除方式。然而，如何使肺癌的切除更加微创始终是胸外科医师的不懈追求。单孔VATS只需一个切口完成手术，与传统多孔VATS相比，能最大程度地减轻胸壁损伤，在减轻术后切口疼痛和胸壁感觉异常方面有明显的优势。近年来，单孔VATS已开始应用于肺癌的切除，并取得不错的临床效果。本文就单孔VATS肺癌切除的进展进行简要小结，单孔VATS是微创肺癌切除的再次升华。

## Abstract (en)

Micro-invasive thoracic surgery, especially represented by video-assisted thoracic surgery (VATS), has become the mainstream of lung cancer resection. Traditional multi-portal VATS techniques, including four-port, three-port, and two-port VATS, have been widely used to perform nearly all kinds of lung cancer resections. However, how to make lung cancer resection less invasive is always the subject that all thoracic surgeons never stop pursuing. Compared with multi-portal VATS, uniportal VATS causes less postoperative pain and paresthesia because only one small incision is made and one intercoastal space is involved. In recent years, good clinical results have been obtained from uniportal VATS in lung cancer resections. In this paper, we'd like to present a brief summary about the progresses made in the application of uniportal VATS in lung cancer resection. Uniportal VATS is a sublimation of micro-invasive lung cancer resection.

目前，肺癌的切除可通过传统开胸、小切口开胸、电视胸腔镜手术（video-assisted thoracic surgery, VATS）、机器人手术等不同入路与方式完成。VATS肺癌切除术极大地减少了传统开胸手术的创伤，并发症发生率、死亡率更低，术后疼痛程度轻且缓解快，对肩关节功能影响小，更好地保护了肺功能，减少了术后创伤因子的产生，更好地保护了免疫功能，患者对术后辅助化疗耐受性更好，明显改善了患者术后的生活质量^[1-4]^。同时，大量研究^[3]^表明胸腔镜肺癌切除在远期生存方面并不亚于传统开胸手术，甚至更优。以VATS为代表的微创手术已成为肺癌切除的主流与共识。传统多孔VATS（包括四孔、三孔及两孔）已覆盖了几乎所有肺癌切除方式。然而，如何使肺癌的切除更加微创始终是胸外科医师的不懈追求。自2004年Rocco等^[5]^首次报道单孔VATS（uniportal VATS）肺楔形切除经验以来，单孔VATS在肺部疾病诊治的适应症已得到不断扩大。单孔VATS只需一个切口完成手术，与传统多孔VATS相比，能最大程度地减轻胸壁损伤，在减轻术后切口疼痛和胸壁感觉异常方面有明显的优势^[6-8]^。近年来，单孔VATS已开始应用于肺癌的切除，并取得不错的临床效果，本文就单孔VATS肺癌切除的进展进行小结。

## 1 单孔VATS的特点与优势

经典三孔VATS的切口布局遵循“棒球场”或“三角形”原则；在操作方面，器械分别经不同操作孔从不同方向、角度到达靶区进行操作；操作时对“三维”与“二维”的转换要求较高。而单孔VATS只有一个操作孔，胸腔镜及所有操作器械均经一个切口进入，这使各器械相互干扰较大，操作角度受限，在使用传统器械时“箭头效应”明显，这些影响因素使单孔VATS复杂手术开展难度增大。但单孔VATS在操作方面亦有其自身特点与优势：①视野与器械在同一投射面，保留了视觉纵深性；②视觉与操作在同一矢状面，更易判断操作距离；③操作支点从胸壁移往胸内，在更接近于靶区的地方形成“操作三角”，更接近于传统开胸操作^[9]^。

## 2 单孔VATS肺癌切除进展

单孔VATS在其开展之初更多地是被应用于一些较为简单的胸部疾病诊断和治疗，如气胸、交感神经链切断术、肺楔形切除、脓胸、外伤及各类活检手术等^[10]^。而对于更为复杂的手术操作（如肺癌切除）却一直鲜有尝试。直至2011年，才由西班牙的Gonzalez^[11]^首次报道了单孔VATS肺叶切除及系统淋巴结清扫（左下肺典型类癌）。此后，作为单孔VATS肺癌切除的先行者，Gonzalez陆续报道了更为复杂和难度更高的单孔VATS肺癌切除，如肺段切除^[12]^、全肺切除^[13]^、肺叶切除+肺动脉成形^[14]^、肺叶切除+部分胸壁切除^[15]^、Pancoast瘤切除^[16]^、支气管袖式成形肺叶切除^[17]^、支气管肺动脉双袖式成形肺叶切除^[18]^等。至此，单孔VATS已基本覆盖了肺癌切除的各类主要术式。近几年，全球各地胸外科专家们亦在陆续开展单孔VATS肺癌切除。2013年新加坡Tam等^[19]^报道了38例单孔VATS肺叶切除（31例肺癌），其中6例（16%）中转开胸，32例成功案例的中位手术时间为216 min。2014年台湾刘家全等^[20]^报道了87例单孔VATS肺癌切除（63例肺叶切除，24例肺段切除），3例（3.45%）中转为两孔VATS。迄今最大宗的单孔VATS肺癌切除病例队列是由Gonzalez-Rivas等^[16]^报道的222例，中转为两孔VATS或开胸的比例为3.6%，平均手术时间为（151.7±76）min。近期，台湾的Wang等^[21]^首次对比了单孔VATS与传统多孔VATS，他们采用倾向性配对法平衡了两组基线资料，发现两组在住院时间、并发症发生率方面无明显差异，而单孔VATS手术时间更短（*P*=0.029），术中出血更少（*P*=0.017），清扫淋巴结更多（*P*= 0.032）。这些经验表明，单孔VATS肺癌切除在技术上是安全可行的，且对于单孔VATS经验较为成熟的胸外科医师来讲，其短期临床效果并不亚于传统多孔VATS。由于单孔VATS开展时间尚短，目前患者长期生存情况正处于随访过程中。

## 3 单孔VATS肺癌切除操作技术的初步经验与共识

和其它任何新技术的开展应用一样，单孔VATS肺癌切除同样存在学习曲线的问题。但目前并无专门探讨其学习曲线的相关报道。现在开展单孔VATS肺癌切除的胸外科医师均拥有丰富的传统多孔VATS的经验。此外，由于单孔VATS通过在同一矢状投射面操作，手眼在同一平面，类似于传统开胸操作模式，因此也有学者^[22]^认为，既往无足够传统多孔VATS经验的外科医师也可以由传统大开胸或小切口开胸直接过度到单孔VATS。大多数医疗中心在单孔VATS肺癌切除方面均尚处于摸索和经验积累阶段，操作技术上也因各外科医师习惯而各不相同，各具特色。纵然如此，目前还是累积了一定经验并逐渐形成一些共识，包括以下几方面：①体位：侧卧位，大多数主刀医师站在患者腹侧，但也有个别医师站在患者背侧进行操作^[22]^。②切口：Gonzalez-Rivas^[16]^采用经腋前线第5肋间切口操作，新加坡Tam等^[19]^采用经腋前线第4-6肋间切口操作，台湾刘家全^[20]^和香港Ng^[23]^等除左上叶外（第5肋间）一般采用经腋前线第6肋间切口操作。总体来讲，切口一般均部于侧胸壁靠前位置（腋前线-腋中线），第4-6肋间，切口大小一般不超过5 cm。因为此处胸壁肌肉层次少，肋间隙较宽，距离肺门纵隔相对较远，有更多空间利于器械对肺门、纵隔结构的处理。③镜头：Gonzalez-Rivas^[16]^、刘家全^[20]^、Tam^[19]^等采用的是5 mm或10 mm 30°镜；Ng等^[22, 23]^则使用的是10 mm 120°镜，认为120°镜可提供更为全景的视野，有利于观察器械头端的位置，操作更加安全。国内胡坚等^[24]^报道了可弯曲镜头的使用。无论选择哪种镜头，均必须保证可以获得不同角度的视野，实现全景监视，赋予操作者更大的操作自由度。④器械：由于切口限制，单孔VATS的器械一般要求细长、多关节或带拐角，既节省切口空间，减少相互“打架”，避免操作时“箭头效应”，又有利于在靶区形成“操作三角”。切割闭合器通常选取带旋转头设计为佳。⑤操作：通常需根据不同操作需求将腔镜与各操作器械灵活换位以保证视野与操作的协调。单孔VATS肺癌切除过程中最棘手的部分在于腔内切割闭合器的放置，需掌握好放置角度以避免误伤和非垂直切割造成残端“猫耳朵”形成。因此，利用带旋转头腔内切割闭合器处理较大血管及支气管是较合理的方法。同时，又需尽量减少切割闭合器依赖，对于较小血管，可选取Hem-o-lock、钛夹或丝线结扎处理，以减少角度不充分的情况下勉强放置切割闭合器引起重要组织损伤的风险。具体到各肺叶切除，Gonzalez-Rivas和刘家全等分别详细地介绍了他们的具体操作模式，并无固定套路，通常依据术中具体情况而选择不同的解剖顺序。但归纳起来其实也就两大模式：“经肺裂操作模式”和“避开肺裂模式”。据Gonzalez-Rivas的经验，双肺下叶切除操作难度相对较小，而左上肺叶切除为所有肺叶切除中最难的，因此建议初学者尽量先从下叶切除开始尝试。至于淋巴结清扫，隆突下淋巴结是较为困难的，Gonzalez-Rivas^[16]^通常选择借助剥离子等硬质器械协助暴露，而刘家全等^[25]^更偏好采用“牵引带”协助暴露，总之，良好暴露是完整清扫的关键。

## 4 四川大学华西医院经验

四川大学华西医院也开展了单孔VATS肺癌切除。在开展肺癌切除前，我们并未像西班牙Gonzalez、台湾刘家全等学者那样事先积累了单操作孔（两孔）VATS操作经验，基本上是直接由经典三孔VATS过渡到单孔VATS，但是在进行复杂单孔肺癌切除之前，我们已有多年单孔VATS简单手术的经验（如单孔肺大疱切除、肺楔形切除、纵隔肿瘤切除、手汗症手术等）。我们通常将切口位于腋前线与腋中线之间第五肋间，一般3 cm-5 cm，采用5 mm 30°镜、双关节腔镜环钳、带拐角金属吸引器、电凝钩、超声刀、带旋转头的腔内切割闭合器、Hem-o-lock等。在肺切除操作方面仍然沿袭了“单向式”的理念；采取“吸引器—电凝无血化游离”减少器械使用，从而减少器械反复进出及过多器械使用导致的相互“打架”；淋巴结的清扫仍采用“无抓持整块纵隔淋巴结清扫法”，除吸引器及能量器械（电凝钩或超声刀）外无需多余器械参与暴露。此外，鉴于单孔VATS的更加“微创”性，我们将之用于双侧同时性多原发性肺癌的诊断及治疗，开展了国际上首例“单孔VATS同期手术治疗双侧同时性多原发性肺癌”，摸索了双侧同时性多原发性肺癌“微创诊治一体化”新的思路与方法。

## 5 小结

肺癌切除经历了传统开胸到小切口开胸，到VATS辅助小切口，再到完全VATS，完全VATS则由多孔逐渐发展至单孔。单孔VATS正在世界范围内呈现星火之势，胸外科医师开始尝试开展单孔VATS肺癌切除。当我们已具备一定单孔VATS肺癌切除经验的时候再回顾过去，单孔VATS虽是传统VATS的进一步延伸，在形式上继承了传统多孔VATS视频操作的特点，但在操作理念上它与传统多孔VATS差别很大，又回归到了开胸手术的操作理念。这样一种发展历程，体现的是肺癌切除术式的不断创新和胸外科医师对微创的极致追求，又是肺癌外科切除理念的传承与回归。单孔VATS无疑是微创肺癌切除的再次升华。相信，随着经验的积累和更合适器械的研发，单孔VATS肺癌切除将会在越来越多的医疗中心开展并被更多胸外科医师所接受和掌握，成为燎原之势。
